# Climate indirectly modulates tree survival of spruce beetle attacks via effects on constitutive and induced secondary metabolites

**DOI:** 10.3389/fpls.2026.1801237

**Published:** 2026-04-22

**Authors:** Ehsan Khedive, Saeideh Fathi Moghanloo, Thomas Seth Davis

**Affiliations:** 1 Department of Forest & Rangeland Stewardship, Warner College of Natural Resources, Colorado State University, Fort Collins, CO, United States; 2 Graduate Degree Program in Ecology, Colorado State University, Fort Collins, CO, United States; 3 Department of Biology, College of Natural Science, Colorado State University, Fort Collins, CO, United States

**Keywords:** bark beetle, Engelmann spruce, herbivory, insect outbreak, monoterpenes, plant resistance

## Abstract

Despite many years of research, it remains challenging to determine why some trees survive bark beetle attacks while others do not. Current theory suggests that survival is predicated by interactions between tree defense systems and climatic drivers, but such interactions are poorly resolved at the scale of individual trees. Using a widespread conifer species (Engelmann spruce, *Picea engelmannii*) and a lethal phloem-feeding herbivore (North American spruce bark beetle, *Dendroctonus rufipennis*) as a study system, we characterized tree responses to mass attack across five populations representing a climate gradient in a field experiment. Over the course of a growing season, we measured variation in monoterpene concentrations in the phloem of 100 trees, half of which were challenged with mass attack using pheromone baits. Study trees were visited two years after baiting to record survival, and match survival to constitutive and induced monoterpenes. Our results revealed that regional climate correlated with constitutive, pre-attack concentration of monoterpenes in spruce phloem: a 1 kPa increase in vapor pressure deficit correlated with a doubling of monoterpene concentration. Higher constitutive phloem monoterpene concentrations predicted tree survival: a one-fold increase in concentration was associated with a three-fold increase in the odds of survival. Regional climate did not significantly affect the magnitude of induced response; however, early induction of monoterpenes in phloem distal to attack sites was associated with an average of twofold increase in survival odds, suggesting rapidity of monoterpene induction is a key resistance trait. These findings extend our understanding of climate-defense relationships in this system and indicate that Engelmann spruce tree populations in warmer and drier sites may have traits associated with higher constitutive resistance.

## 1 Introduction

Forest mortality from bark beetles has consequences for global carbon budgets (Kurz et al., 2008), biodiversity (Beudert et al., 2015; Davis et al., 2020; Plath and Fischer, 2024; Vrba et al., 2024), and the provisioning of ecosystem services including both timber resources and recreation economies (Morris et al., 2018; López-Andújar Fustel et al., 2024). In western North America, Engelmann spruce (*Picea engelmannii* Parry ex Engelm.) is a common component of high-elevation forests, forming large contiguous stands throughout the Rocky Mountains. In recent decades, outbreaks of the North American spruce bark beetle (*Dendroctonus rufipennis* Kirby, Coleoptera: *Curculionidae*) have caused extensive mortality in Engelmann spruce forests (Dymerski et al., 2001; Potter and Paschke, 2024) resulting in up to 100% mortality in some areas (Rodman et al., 2021) and the death of millions of trees. Various recent studies (e.g., Sherriff et al., 2011, Hart et al., 2014, 2017, Pettit et al., 2020, and others) indicate that climate anomalies have preceded these mortality events, but the linkage between climate variation and tree defense physiology in response to *Dendroctonus rufipennis* attack remain poorly understood. However, climate-mediated shifts in tree defensive capacity in response to bark beetle attack could partially explain how and why these outbreaks occur following climate anomalies such as drought or elevated temperature.

Climate conditions have dynamic impacts on forest insect populations (Bracalini et al., 2024). As ectotherms, populations of most bark beetles are highly responsive to subtle climatic shifts, which may affect flight phenology, synchrony, and development rates (Dell and Davis, 2019; Bentz et al., 2022; Johnson and Haynes, 2023; Bracalini et al., 2024). Additionally, isolated climate-related events such as storms, wind events, and avalanches can trigger local infestations of *D. rufipennis* by direct physical damage to trees, which may cause larger ecosystem-level disturbances leading to rapid colonization of the downed trees in the ecosystem (de Groot et al., 2018; Karpov et al., 2024).

In addition to these direct effects, a changing climate will increase physiological stress for some tree species (Taccoen et al., 2022). Elevated environmental stress has consequences for tree resistance to bark beetles (Herms and Mattson, 1992; Choudhary and Senthil-Kumar, 2022), and may reduce tree vigor and the functionality of defense systems (Raza and Bebber, 2022). Quantitative resin-based defenses are a primary conifer resistance factor against bark beetles (Denham et al., 2019; Davis, 2020; Mullin et al., 2021; Al-Khayri et al., 2023; Baker et al., 2024), and usually consist of terpenoid-based secondary metabolites including mono-, sesqui-, and diterpenes (Phillips and Croteau, 1999) that are exuded from resin ducts in tree phloem and sapwood (Seifert et al., 2010). Studies indicate that monoterpene constituents of Engelmann spruce phloem are toxic to *D. rufipennis* and associated fungi carried by the beetle, regardless of the blend (Davis et al., 2018; Davis, 2020). Terpenoids are constitutively present in spruce needle, phloem, and xylem tissues as pre-formed defenses, but can also be induced in response to biotic challenge by insects and pathogens (Celedon and Bohlmann, 2019). This is usually reflected by an increase in their concentrations in affected tissues. Yet, terpenoids are also synthesized in response to high temperature and low water availability to stabilize cell membrane structure by scavenging reactive oxygen species (ROS) (Qaderi et al., 2023), suggesting dual functionality in tolerance towards both biotic and abiotic stresses (Qaderi et al., 2023; Ogwu et al., 2025; Rabeh et al., 2025). Few studies have also suggested that under water and nutrient limitation, plants may divert excess fixed carbon into secondary metabolism (Prescott, 2022).

Although previous studies attribute Engelmann spruce mortality from *D. rufipennis* to climatic conditions, which may be driven through the direct and indirect mechanisms described above, there are presently no studies that link these patterns with specific physiological process in an experimental context. Here, we present a field experiment designed to investigate variation in Engelmann spruce monoterpenes, as a chemical defense, in response to mass attack by *D. rufipennis* and match this variation to subsequent tree survival, interpreting constitutive and inducible defenses along a climatic gradient. We test the following specific hypotheses: i) regional precipitation, evaporative stress, and temperature modulate constitutive and induced monoterpene concentrations in spruce; ii) constitutive and induced monoterpene concentrations predict tree survival of mass attack; and iii) the rapidity of induction is a more important predictor of survival than overall magnitude. By manipulating pheromone-mediated mass attack behaviors, we experimentally incited colonization in spruce populations both in pre- and post-outbreak forests. Our findings enhance our understanding of how long-term local climate shapes tree defense capacity against phloem-feeding bark beetles, providing a necessary foundation for interpretation of tree response to short-term, climate change-driven stressors.

## 2 Materials and methods

### 2.1 Study system and site description

The study took place in alpine forests of north-central Colorado. Engelmann spruce forests cover a significant portion of the southern Rocky Mountains in Colorado (Alexander and Shepperd, 1990).This species is considered a high-elevation species and typically defines the upper forest timberline (upwards of 3500 m) in the southern extent of its range in the Rocky Mountains. At lower- and mid-elevation ranges (2500–3500 m) it often occurs in mixed stands with lodgepole pine (*Pinus contorta* Dougl. ex. Loud.) and subalpine fir (*Abies lasiocarpa* (Hook) Nutt.).

**Figure 1:**
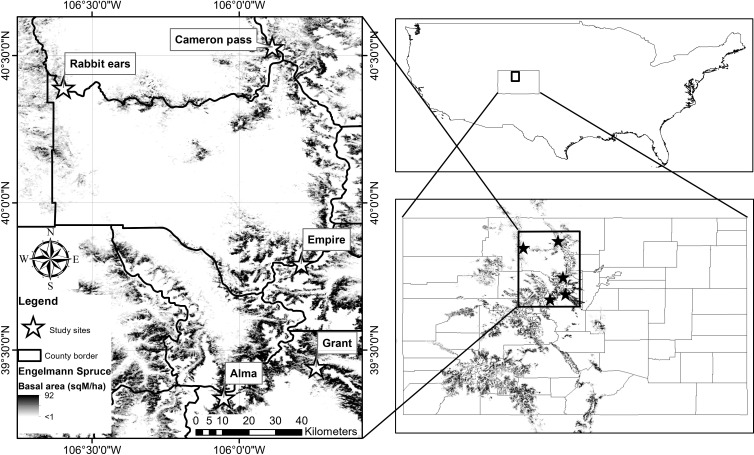
Geographical location of study sites in stands of Picea engelmannii in Colorado.

We selected five sites among fourteen where the phloem monoterpene composition of Engelmann spruce was previously described (Davis et al., 2018), choosing sites to cover the highest range of vapor pressure deficits (VPD), temperature, and precipitation among the others. The study stands were in the proximity of Cameron pass (post outbreak), Rabbit ears pass (pre-outbreak), Alma (endemic phase), Grant (endemic phase), and Empire (endemic phase) (**Figure 1**). Ten-year (January 2014 - December 2023) monthly climate data (precipitation, mean temperature, maximum vapor pressure deficit) were extracted for study sites from the standard 4km grid explorer in the PRISM climate model (**Table 1**) for use in site classification and analyses.

**Table 1:** Location and 10-year climate characteristics (2014-2023) of study sites based on PRISM climate data.

Site	Coordinates	Elevation (m)	Average annual temperature (C)	Average daily max VPD (kPa)	Average annual precipitation (mm)
Cameron pass	40.512, -105.887	3100	1.15(7.7)	0.81(0.54)	1055(136)
Rabbit ears pass	40.390, -106.608	2800	3.6(8.5)	1.13(0.77)	762(100)
Alma	39.337, -106.052	3350	1.06 (7.8)	0.8(0.5)	749(122)
Grant	39.450, -105.742	2950	2.84(7.6)	1.03(0.58)	413(68)
Empire	39.775, -105.798	3000	0.9(7.8)	0.68(0.39)	911(96)

Values in parentheses represent standard deviation.

### 2.2 Tree baiting experiment

In each study site, n=20 healthy, mature, dominant and codominant spruce trees were selected for study. Of these, half were randomly selected for baiting, the remaining trees were considered as controls, for a total of n=100 study trees distributed across five sites (average diameter of 45.6 ± 2.2 SE cm) (**Table 2**). Pheromone baits for *D. rufipennis* containing a proprietary blend of frontalin, seudenol, and spruce kairomones (Synergy Semiochemicals, Delta, Canada; Catalogue #3289) were used for inciting mass attacks on selected study trees. Baits were applied on June 10, 2022 at 2m height on random aspect of the trunk. Pheromone packets were left on baited trees during the sampling period and were removed in September 2022, and the phloem of all study trees (both control and treated) were sampled at roughly 10-day intervals during this period (sampling details and processing described in more detail below).

**Table 2:** Stand density (sqm/ha) characteristics of the studied sites according to USFS database along with the diameter of studied trees in centimeters.

Site	Spruce basal area	Spruce dominance	Average diameter (SD)
Cameron pass	11.3	57%	60.7 (38.4)
Rabbit ears pass	12.9	38%	43.4 (12.9)
Alma	27.3	82%	40.5 (11.5)
Grant	28.9	75%	30.5 (6.6)
Empire	28	66%	52.6 (10.6)

We returned to baited trees in September 2024 to record survival. Entry hole (i.e., ‘pitch tube’) density was also measured on each baited tree at this time within two 30 × 30 cm sampling windows located on the south and north sides of the stem as an estimate of colonization pressure. Following bark beetle attack, trees face three distinct fates. Some successfully defend themselves by expelling attacking beetles before galleries are established. Others experience partial colonization, where beetles occupy limited sections of the bole in so-called strip attacks. In contrast, heavily attacked trees typically succumb within one to two years. Notably, partially colonized trees occupy a unique ecological role: they remain alive while simultaneously sustaining beetle populations, effectively bridging host survival and insect persistence. Hence, baited trees were categorized as ‘beetle-killed, ‘survived-colonized, and ‘survived-uncolonized’. Successful colonization was inferred from bark removal by woodpeckers, observed two years after the baiting experiment. We classified trees as ‘dead’ if they had either completely shed their needles or exhibited full crown browning by September 2024.

### 2.3 Phloem sampling and processing

Phloem sampling was started on June 10, 2022, when the baits were applied on the trees. Sampling was repeated every 10 days until day 210 and every 15–20 days until day 260 (September 21, 2022), for a total of 9 sampling rounds. All the 100 trees were sampled in every sampling round. At each sampling round, tree phloem was collected from the study trees using two 7 mm circular punches (5 cm apart horizontally) taken semi-systematically at a height range of 0.5 meter above and below breast height, leaving an average of 10cm of vertical space between the punches plus random tangential spacing. It was impractical to take punches according to an exact systematic pattern as there were numerous sources of variation on the bark, especially random location of bark beetle attacks. After excision from trees, each phloem sample was immediately transferred to a dewar flask containing liquid nitrogen (in the field) and returned to the lab for monoterpene analysis. This sampling scheme resulted in a total of n=900 phloem samples collected (100 study trees × 9 sampling rounds). As both treated and control trees were sampled similarly during the experiment, effects of repeated sampling was also adjusted for.

Upon return to the lab, phloem samples were stored in a -80 °C freezer until analysis. Samples were prepared for chemical analysis by pulverizing 100–120 mg of fresh phloem using liquid nitrogen in mortar and pestle, and extracts were made by adding 200 µl of HPLC grade chloroform (Millipore-Sigma, MA, United States) and vortexing for 30 s in 1.5 ml microcentrifuge tubes. We used chloroform instead of hexane because it yielded better extraction results (single wash and more monoterpenes, data not shown). An internal standard (n- decane, 20nl/ml concentration) was added to the chloroform and used for quality control purposes. Thereafter, the tubes were centrifuged at 10,000 rpm for 1 min and the infranatant were transferred to GC/MS autosampler vials for analysis using a pipette. High purity (>97%) monoterpenes were purchased from Sigma-Aldrich (Millipore-Sigma, MA, United States) as analytical standards. We used available external monoterpene standards as surrogate compounds for quantification because not all monoterpene standards were obtainable in the lab (refer to supplementary R Markdown file for details). Monoterpenes with similar structures tend to exhibit comparable response factors, as supported by our own data (not shown) and by the findings of Szulejko and Kim (2014).

Extracts were analyzed using an Agilent 7820A gas chromatograph (GC) coupled with 5977B mass selective (MS) detector (Agilent Technologies, Inc., Santa Clara, USA) outfitted with a non-polar DB-5 column (30m length, 0.25 mm ID, 0.25 µm film thickness) for analysis. The temperature program was as follows: 1 minute at 45 °C, then increase to 240 °C at 7 °C/min, then hold for 1 minute, then increase to 270 °C at 20 °C/min and hold for 5 minutes. The inlet was operated in splitless mode with 1ml/min helium flow at temperature of 260 °C and aux heater at 280 °C. MS source and quadruple temperatures were constant at 230 °C and 150 °C respectively. The injection volume was 2 µl. The scan range for MS was put to 31–400 Da with a threshold of 150.

After GC/MS analysis, we acquired and processed sample chromatograms using MassHunter Qualitative Analysis (Agilent Technologies, Inc., Santa Clara, CA) to run raw spectra against MS libraries (NIST 2014, Wiley 10^th^ ed.). All detected compounds were directly exported to a csv file and imported to R software for further data handling and statistical analysis.

### 2.4 Statistical analysis

All data handling and analysis were done using R (R Core Team, 2024) and R Markdown (Baumer and Udwin, 2015). We expressed compound concentrations based on fresh phloem weight (rather than dry weight), as bark beetles feed on fresh tissue and therefore, this measure is ecologically more relevant. Before any statistical analysis we used Winsorization to remove outliers larger than the 99th percentile and reduce false positive results in fold-change calculations (Yang et al., 2024). For the purpose of statistical analysis for baited trees we divided the dataset into flight period (days 160 – 190) and post-flight period (days 200-260) according to flight period data in Colorado from Davis and Hansen (2018).

In general, concentrations of all detected monoterpenes exhibited similar seasonal patterns, characterized by a mid-season peak and subsequent decline (**Supplementary Figure 1**), indicating strong correlations among compounds (**Supplementary Figure 2**). Therefore, all the statistical analysis was done on total concentration of detected monoterpenes rather than individual monoterpenes. Fold-change values were obtained by dividing the concentration measured in baited trees by the corresponding mean concentration of control trees at the same site and sampling date, thereby producing values above zero. To address the hypothesis that “climate variables modulate constitutive and induced monoterpene concentrations in spruce”, we used a complete-block linear regression model. Site-specific climate parameters were treated as explanatory variables, sampling round as a blocking factor, and monoterpene concentrations were log-transformed prior to analysis (i.e. log-linear regression). We applied this model to the natural logarithm of monoterpene concentration to test whether climate variables (mean annual temperature, maximum annual vapor pressure deficit, and annual precipitation) explain variation in total constitutive monoterpene concentrations in control trees.

To evaluate induced responses, we used the same set of site-specific climate variables in a complete-block regression model with fold-change as the response variable to assess whether climate conditions influence induced monoterpene production during the flight season.

To address the hypothesis “constitutive and induced monoterpene concentrations predict tree survival of mass attack,” we first conducted a complete-block ANOVA to test whether fold-change in concentration differed among trees of different survival status across the entire season. In the second step, we performed logistic model on both during−flight and post−flight datasets, using fold-change at each sampling round as predictor. In these analyses, fold-change on day 160 was treated as the constitutive concentration because trees had not yet been attacked by beetles at that point.

To address the hypothesis “the rapidity of induction is a more important predictor of survival than overall magnitude” we defined speed as the firs sampling time (i.e. day of year) where the fold-change exceeded 1, and magnitude as the mean fold-change across the growing season. A fold-change threshold of 1 was selected under the assumption that control and baited trees exhibit comparable baseline concentrations under similar conditions; thus, values exceeding this threshold reflect induction. This threshold represents a minimally conservative criterion intended to capture the earliest detectable response. Then we defined a logistic model with main and interaction effects of both speed and magnitude. We considered speed effect in the model as factor, not a continuous variable.

## 3 Results

### 3.1 Climate variables modulate constitutive but not induced monoterpene concentrations in Engelmann spruce

**Figure 2:**
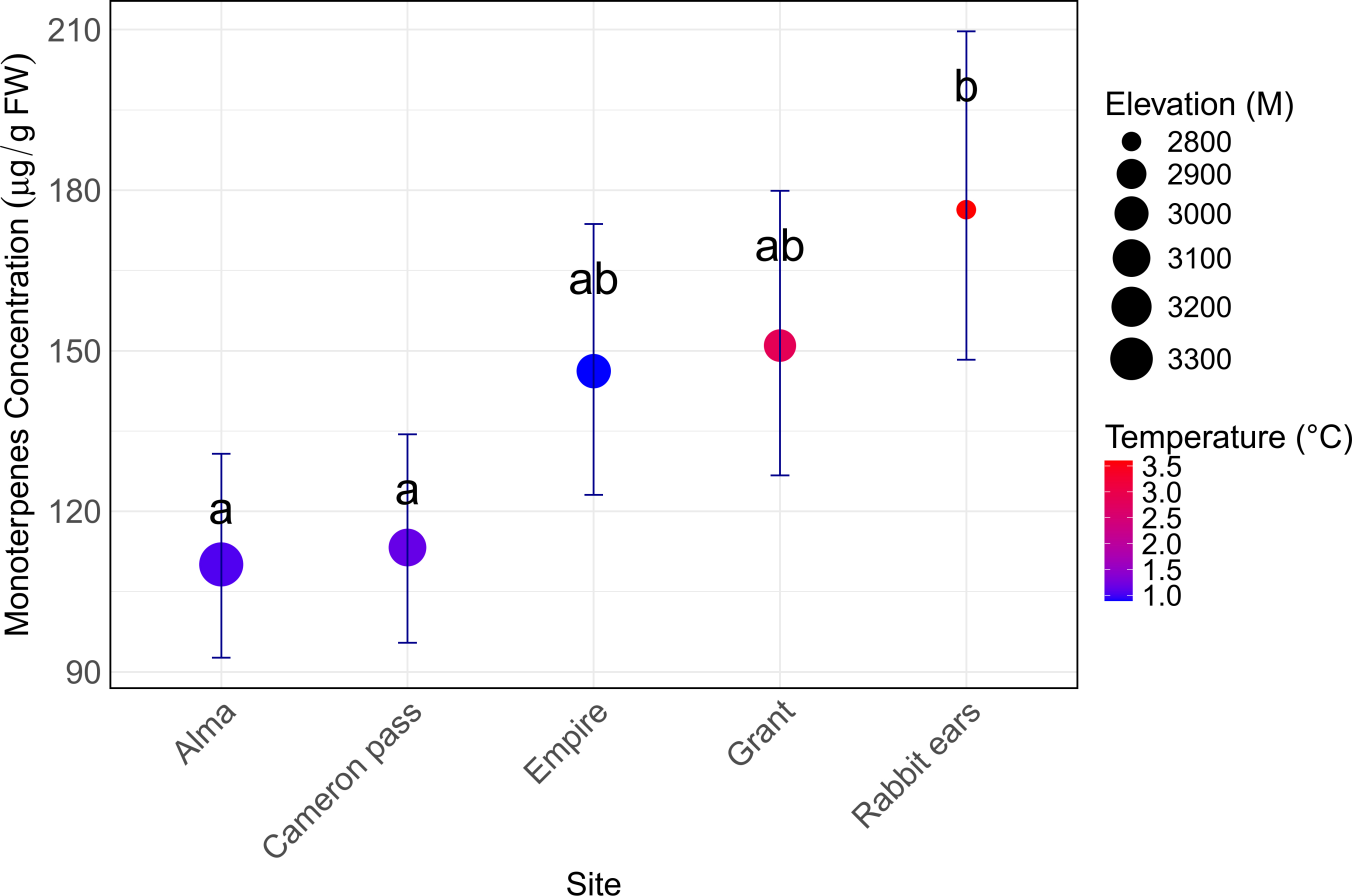
Phloem monoterpene concentration of *Picea engelmannii* control trees in study sites, relative to variation in temperature and elevation. Different letters show significant differences identified by *post hoc* analysis (Tukey’s HSD). Each point on the chart represents an average of 10 biological and 9 time-replicate samples (i.e. n=90 per point).

Sampled spruce populations were associated with different climates, and this was correlated with differences in baseline phloem monoterpene concentrations (i.e. concentration in control trees). Trees at higher altitude sites, where the average annual temperature was lower, had significantly lower mean monoterpene concentrations. Overall, colder, high-elevation sites such as Alma and Cameron Pass had approximately 45% lower monoterpene concentration than the warmer, lower-elevation site Rabbit Ears Pass (**Figure 2**; **Supplementary Table 1**).

**Figure 3:**
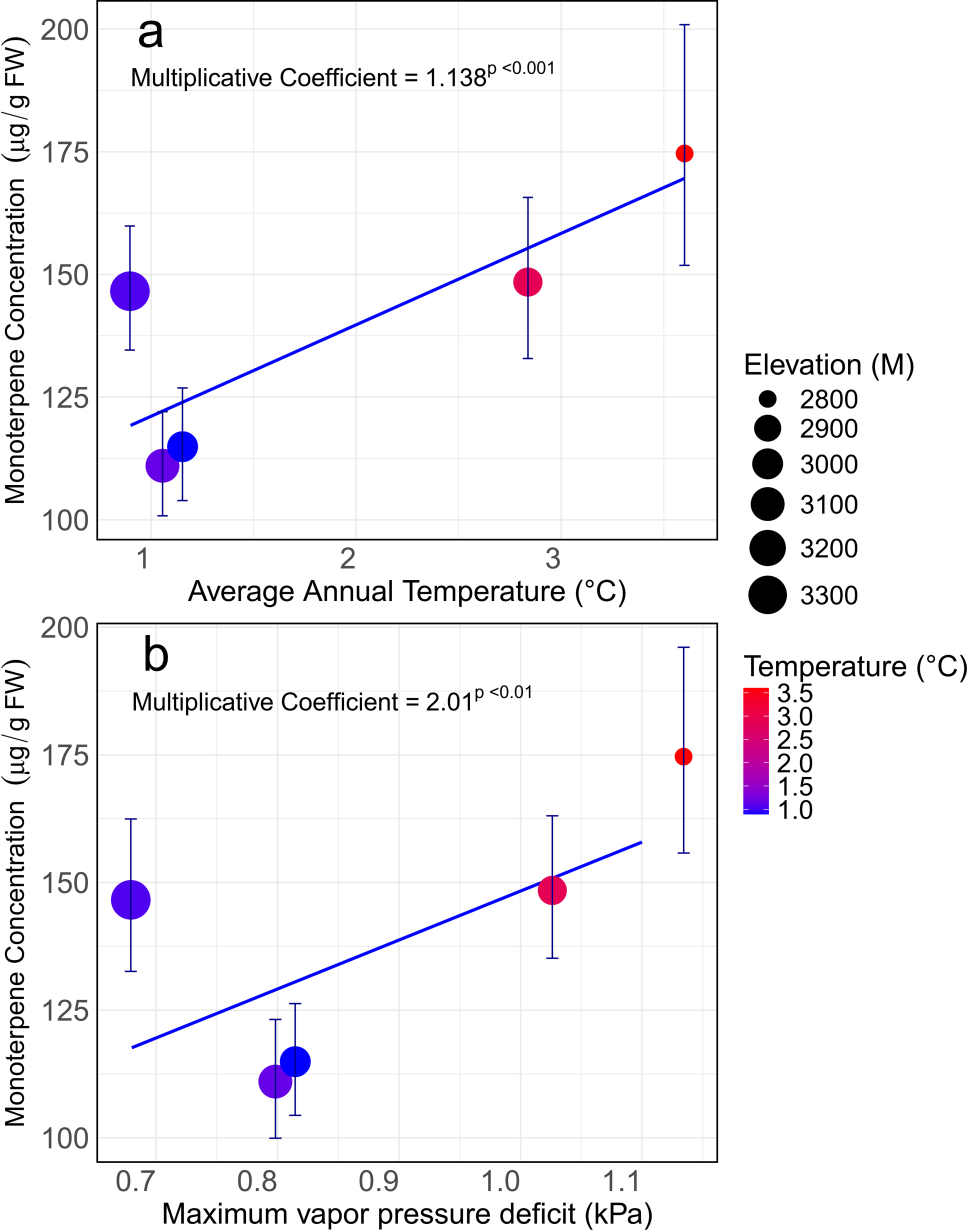
Relationships between phloem monoterpene concentration and site-specific average annual temperature **(a)**, and maximum vapor pressure deficit **(b)** (10-year means) in control *Picea engelmannii* trees. Coefficients were calculated based on a log transformed model. Each point on the chart represents an average of 10 biological and 9 time-replicate samples (i.e. n=90).

Log-linear regression indicated that monoterpene concentrations were correlated with 10-year average climate variables across the different populations. For every 1 °C increase in average annual temperature, the fitted model predicts an increase in constitutive phloem monoterpene concentrations of ~14% (**Figure 3a**; **Supplementary Table 2**). There was not a significant relationship between annual precipitation and mean phloem monoterpene concentrations (p-value = 0.07, **Supplementary Table 3**), though a negative trend was evident. Maximum VPD (i.e., evaporative stress) was also a strong predictor of variation in constitutive phloem monoterpene concentrations; for every 1 kPa increase in maximum VPD, monoterpene concentrations approximately doubled (**Figure 3b**; **Supplementary Table 4**).

Fold−change analysis of induced monoterpene concentrations during and after the flight period indicated that variation in induced monoterpenes was not associated with temperature, despite the patterns observed for constitutive concentrations (**Supplementary Table 5**, only temperature test results are shown).

### 3.2 Constitutive and induced monoterpene concentrations predict tree survival of mass attack

**Figure 4:**
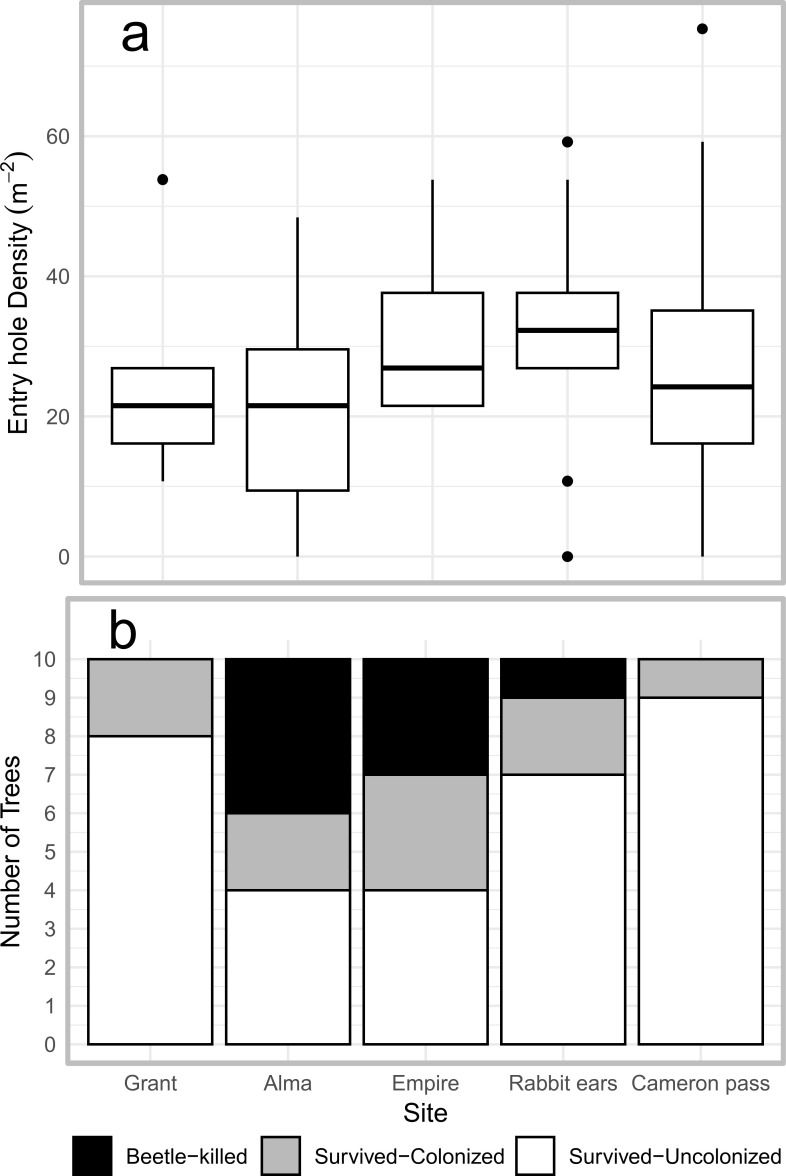
**(a)** Beetle entry hole density on *Picea engelmannii* tree boles per square meter of bole surface (replicates are the sum of survived trees in each site from lower panel), and **(b)** number of killed, colonized, and uncolonized baited trees in each site.

**Figure 5:**
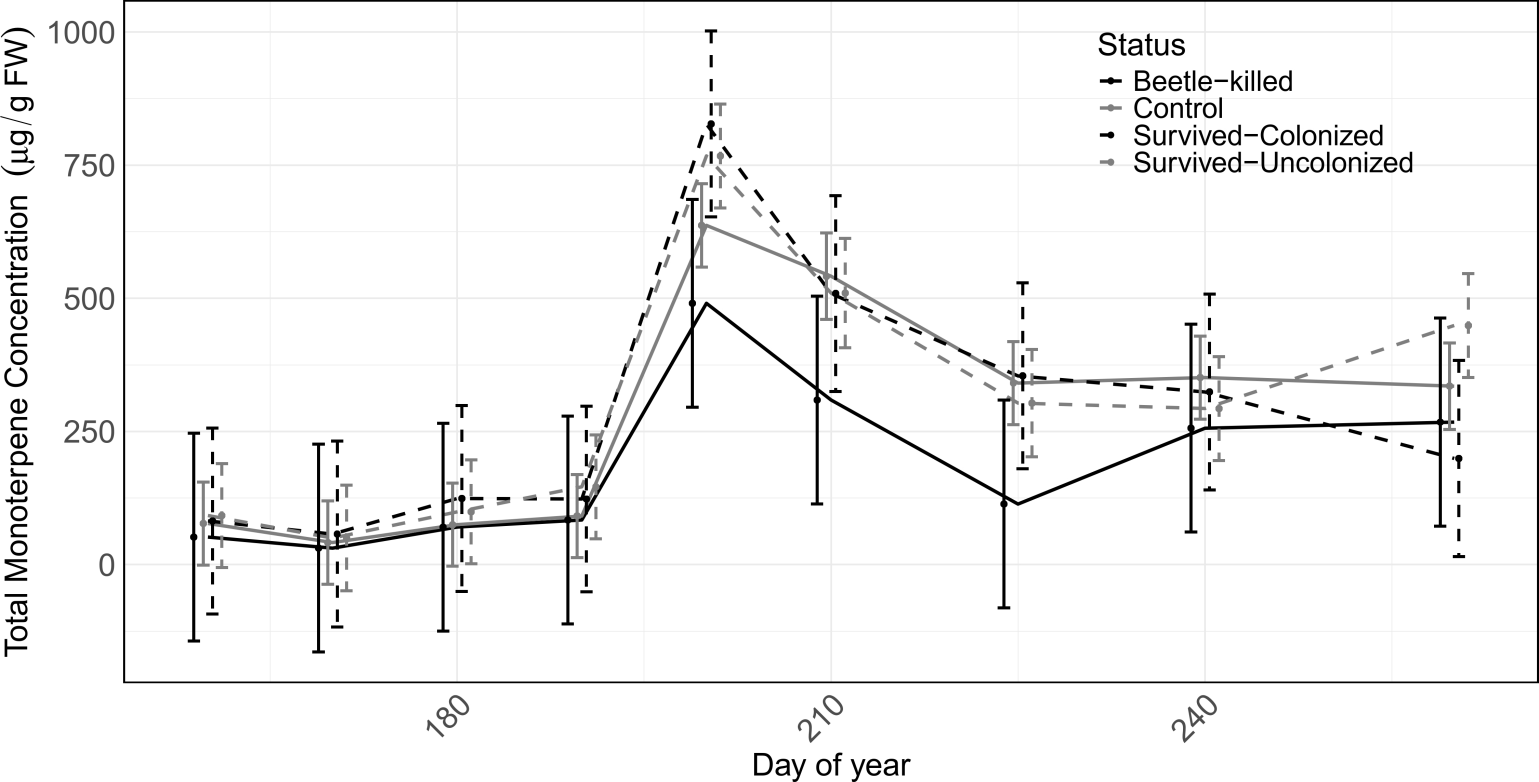
Monoterpene concentration of *Picea engelmannii* phloem in studied trees in all sites during the growing season. Error bars are 95% confidence interval for each sampling day for each status group.

No significant differences were observed in entry hole density between the study sites, indicating similar attack pressure (**Figure 4a**; **Supplementary Table 6**). Mortality was recorded for several trees at Alma and Empire sites, whereas at Rabbit Ears Pass only a single tree died. The other two sites did not experience any mortality over the two-year period (**Figure 4b**), despite pheromone baiting. Testing monoterpene concentration against tree survival revealed that trees that died after two years had lower total monoterpene concentration throughout the season, particularly after flight period, when not adjusted for site effects. In contrast, colonized and uncolonized trees generally had higher concentration compared to non-attacked control and beetle-killed trees (**Figure 5**).

**Figure 6:**
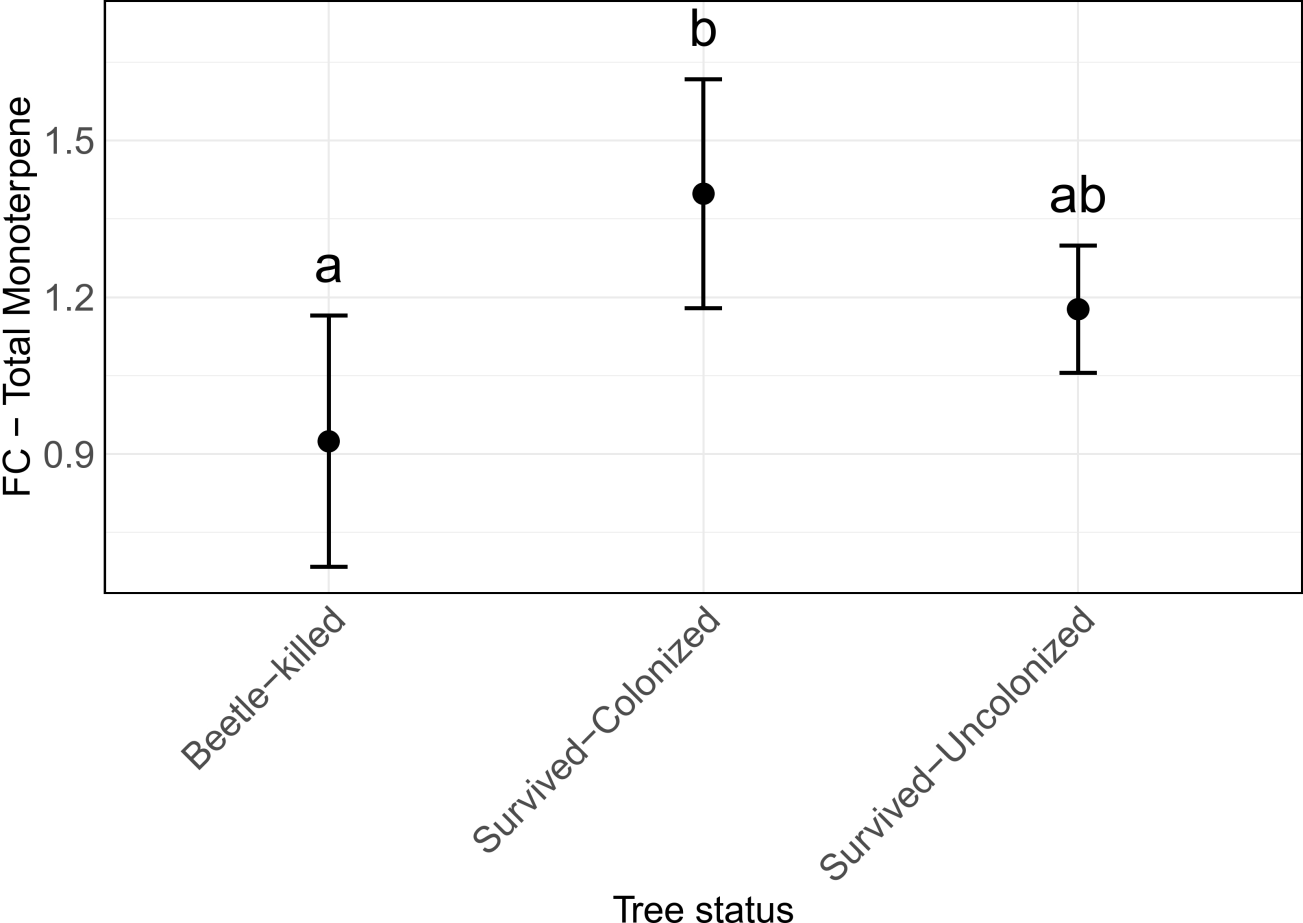
Fold-change in monoterpene concentration in different baited *Picea engelmannii* trees compared to site and location specific control trees. Error bars are 95% confidence interval. Groups with different letters are significantly different at 5% alpha level using Tukey’s HSD test.

In general, the average monoterpene concentration over the whole season in beetle-killed trees were lower than site-specific control trees (approximately 10% lower), while colonized trees that survived attack had about 35% more monoterpenes in their phloem, and uncolonized trees had 20% more (**Figure 6**; **Supplementary Table 7**).

**Figure 7:**
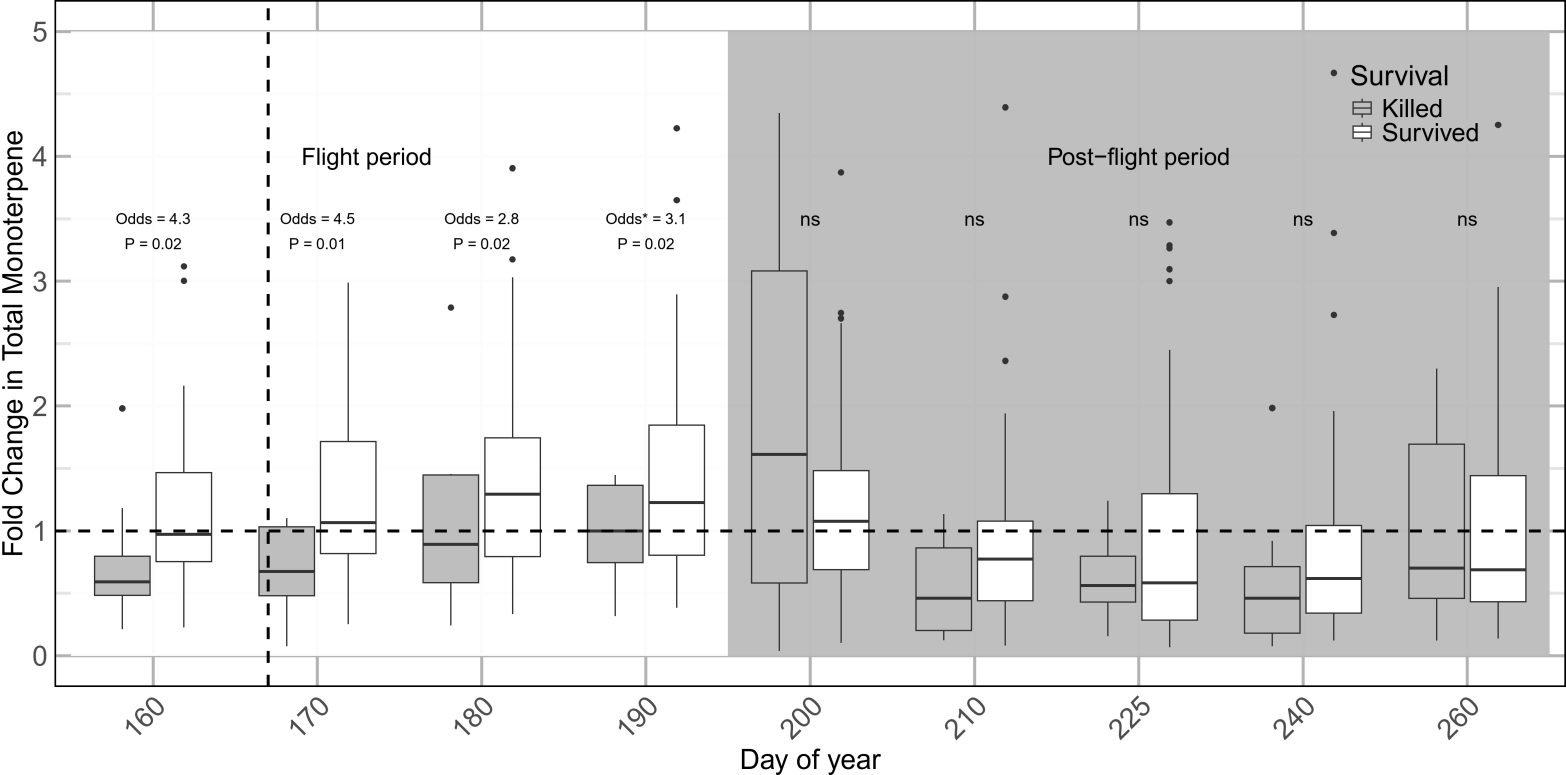
Fold-change in monoterpene concentration in different baited *Picea engelmannii* trees compared to site and location specific control trees during and after flight period. Odds of survival were calculated using logistic regression. Colonized and uncolonized trees were pooled as “survived” trees for the purpose of analysis. Dashed vertical line represents the approximate day of attack initiation (Day 167), and dashed horizontal lire represents the baseline concentration.

To test the effect of fold-change concentration on survival across the entire season we ran two independent logistic models for samples collected during flight period (day of year 160-190) and samples collected after the flight period of the beetles (day of year 200-260) as described in the methods. The models revealed that at the time of the first sampling, on day 160 (before the trees were attacked, i.e., constitutive concentration) fold-change in monoterpene concentrations significantly predicts tree survival. The model showed that for every fold-change in constitutive monoterpene concentration, the chance of survival increased three-fold. Testing fold-change during the flight period also revealed a highly significant effect, indicating that each unit increase in induction fold-change during the flight period was associated with more than a twofold increase in the odds of survival. Post flight changes in monoterpene concentration did not differ significantly between beetle-killed and survived trees (**Figure 7**; **Supplementary Table 8**).

### 3.3 Both the rapidity and magnitude of induction had similar effect sizes

A separate model to independently test the timing and the magnitude of response showed no evidence of a significant difference in the effects of speed or magnitude of monoterpene induction (p-value >0.05, Deviance = 25.88, Null deviance = 39.88) on tree survival (**Supplementary Table 9**).

## 4 Discussion

Terpenes are among the most resource-intensive defense mechanisms in plants (Qaderi et al., 2023). Field studies, particularly on Norway spruce (*Picea abies* L.), have explored various induction methods to stimulate monoterpene production (Zhao et al., 2011; Schiebe et al., 2012; Mageroy et al., 2020), demonstrating their important role in defending trees from bark beetle mass attacks. Here, we assessed tree survival over a two-year period as our primary indicator of defensive capacity. In this system, the timing of the two-year survival assessment is important because North American spruce beetles may initiate galleries in small sections of the bole, causing injury but not mortality in the year of attack (so-called “strip attacks”, Massey and Wygant (1954)), and if successful, subsequently mass-colonize and kill their hosts in the following year. Here, our field experiment shows that pre-formed, constitutive phloem monoterpene concentrations are important for predicting tree survival over a two-year period. Moreover, higher constitutive phloem monoterpene concentrations were found in populations at lower elevations, in warmer conditions, and with higher evaporative stress. This result supports the idea that climate change primarily enhances environmental suitability for bark beetles (Pettit et al., 2020), rather than increasing tree susceptibility through abiotic stress (Hart et al., 2014). Our data suggests that in high-elevation alpine ecosystems, alterations in constitutive defenses, driven by long term regionally drier and warmer climate, may act more in favor of the host tree. Consequently, outbreak patterns observed during past three decades are more likely related to beetle physiology under a changing climate.

This finding contrasts with earlier studies reporting higher monoterpene levels or improved conifer survival at higher elevations in other conifer species (Mullin et al., 2021; Karpov et al., 2024; Thompson et al., 2024). However, research isolating the direct effects of climate conditions often support a positive effect of temperature, and even drought, on terpene production in some conifer species (Gaylord et al., 2007; Wallis et al., 2011). This apparent discrepancy may stem from the ecological niche of Engelmann spruce, a high-elevation species typically found above 2,200 meters elevation and often forming the upper timberline in the Rocky Mountains, exposing the species to highly stressful environment of the cold timberline. Engelmann spruce commonly co-occurs with lodgepole pine, particularly at lower elevations and on southern aspects. Because lodgepole pine is more tolerant of warm, dry conditions, competitive interactions may constrain the distribution of Engelmann spruce in these environments, thereby limiting its exposure to climatic conditions that can impose physiological constraints on metabolism (Stohlgren and Bachand, 1997). While population-level differences could contribute to variability in monoterpene concentration across sites, our results closely track a regional temperature gradient effect. This pattern could reflect temperature constraints on tree physiological processes, including growth and development (Körner et al., 2016), and upregulation of secondary metabolites to stabilize cell function and mitigate oxidative stress under suboptimal thermal conditions (Qaderi et al., 2023; Ogwu et al., 2025). Plants produce secondary metabolites such as monoterpenes as part of their defense and stress-response systems. Under drought and elevated temperatures, these compounds can help mitigate cellular damage (Laxa et al., 2019), often leading to altered concentrations and emissions (Trowbridge et al., 2021). There is also a possibility that the trees divert excess carbon to secondary metabolite pools under resource scarcity (water and nitrogen) (Prescott, 2022). Gaylord et al. (2007) showed that resin flow was higher in *Pinus ponderosa* under high water stress and low photosynthesis capacity. Therefore, multiple biochemical and signaling pathways mediate the induction of secondary metabolites in response to environmental stressors (Akula and Ravishankar, 2011); however, the regulation and relative contribution of these pathways vary among species and needs further study in this species.

We didn’t find a significant effect of precipitation, however a strong, significant positive effect of evaporative stress (i.e. VPD) on constitutive monoterpene concentration in control trees was evident. Elevated VPD was clearly associated with higher monoterpene concentration. Although VPD is often correlated with temperature, the nearly threefold difference in precipitation between sites suggests that evaporative-driven drought stress may directly contribute to increased monoterpene production. This finding contrasts with several previous studies that reported a negative or neutral effect of drought on terpene concentration (Kolb et al., 2019; Erbilgin et al., 2021; Trowbridge et al., 2021). Such discrepancies may be attributed to experimental differences, as most prior studies were conducted on seedlings under short-term, controlled environmental conditions (Blodgett and Stanosz, 1998; Klutsch et al., 2017; Trowbridge et al., 2021), while we looked at the regional microclimate effect on mature trees. Studies on mature trees have also produced mixed results when simulating drought through trenching (Kolb et al., 2019; Erbilgin et al., 2021). This might occur because mature trees respond differently to short-term drought compared to long-term exposure to regional microclimate (i.e. our study). Notably, our findings align with the study done by Gaylord et al. (2007) on *P. ponderosa* and the broader understanding that moderate drought can enhance conifer defenses against bark beetles (Netherer et al., 2024; Singh et al., 2024).

Among the sites, Empire showed a marked deviation from the overall trend in both the temperature and VPD models. However, monoterpene concentrations at this site corresponded closely with its elevational position among the sites. While few studies have documented the influence of elevation on secondary metabolite concentrations in other conifer species, the underlying mechanisms driving this pattern remain unresolved and needs further studies (Mullin et al., 2021).

Although temperature and evaporative stress played a significant role in shaping constitutive monoterpene concentration, there was no evidence of their effect on rapidity or magnitude (fold-change) in monoterpene induction in response to beetle attacks, either during or after the *D. rufipennis* flight period. Nonetheless, attack densities were similar on baited trees across study sites, indicating similar biotic pressure. This differs from results of earlier studies on other conifer species (e.g., *Pinus edulis*), showing that drought can suppress resin induction in response to bark beetles and associated fungal pathogens (Thompson et al., 2024; Malone et al., 2025), leading to reduced resin-based defenses. In Engelmann spruce, traumatic resin ducts may not form until up to a year after biotic challenge (Ott et al., 2021), although we observed the formation in the same year of attack in this study. Thus, the upregulation in phloem monoterpene concentrations we observed in surviving trees during the year of baiting may correspond more to metabolic reserves (e.g., variation in non-structural carbohydrates) than to population- or climate-mediated variation (Erbilgin et al., 2021). In addition, our study populations were selected to reflect long-term adaptations to different microclimatic conditions, whereas many prior studies relied on short-term, acute drought stress to evaluate tree physiological responses to bark beetle challenge. For example, several recent studies (Basile et al., 2024; Netherer et al., 2024) reported no change in either constitutive or induced phloem monoterpene concentrations in response to drought in Norway spruce. Collectively, our results and these earlier studies suggest that neither acute drought stress nor population variation due to long-term exposure to varying aridity conditions have strong effects on the induced defense response of spruces.

Here we observed higher mortality in colder sites (i.e., Empire and Alma); however, no mortality was recorded at Cameron Pass. The lack of mortality may reflect the post-outbreak condition of the site, where remaining trees are presumably more resistant to bark beetle attack or beetle populations are possible insufficient to overwhelm the baited trees. In the case of the mountain pine beetle, several studies have reported that surviving trees tend to have larger or denser resin ducts than trees present before outbreaks (Ferrenberg et al., 2014; Baker et al., 2024). However, this pattern was not observed in our study, as terpene concentrations did not differ between Alma (endemic phase) and Cameron Pass (post outbreak), which were relatively similar in both temperature and VPD.

Across sites, surviving trees experienced different median attack densities and this showed no clear trend in relation to site climate. This indicates that warmer climates did not lead to higher attack rates in our study sites, while tree survival rate varied (range: 60-100% survival). We showed that a onefold increase in constitutive monoterpene concentration corresponded to a significant threefold increase in survival likelihood. Although relatively few studies have focused on constitutive terpene concentrations, our findings support the idea that elevated terpene content, whether driven by warmer, drier conditions (similar to *P. ponderosa* as in Gaylord et al. (2007)) or by prior outbreaks (Siegert et al., 2024), can enhance resistance and reduce mortality during subsequent beetle attacks.

We showed that early induction was also important as a defense against *D. rufipennis* attack. Although the model testing response time and magnitude independently was not significant (partially due to reduced degrees of freedom), indicating no difference in effect size between these two components, the difference between model deviance and null deviance (25.88 vs 39.88) shows that the timing and magnitude defines roughly 35% of tree survival. Additionally, the time-dependent model revealed that higher monoterpene concentrations during the flight period significantly increased tree survival. An average onefold increase in monoterpene concentration during the first month following attack was associated with an average twofold increase in the odds of survival. Previous studies have emphasized the importance of inducible responses in bark beetle defense at the site of attack (Thompson et al., 2024). Here, we showed that the induction of defensive compounds distal to infected tissue can reach concentrations critical for tree survival. Interestingly, monoterpene concentrations declined markedly following the flight period in both surviving and beetle-killed trees, falling below control baseline levels. While elevated monoterpene production during the flight period likely reflects stress-induced upregulation of defensive secondary metabolism (Keefover-Ring et al., 2016; Musso et al., 2023), the subsequent decline may indicate post-stress resource limitation, whereby reduced carbon assimilation constrains continued investment in monoterpene biosynthesis (Thompson et al., 2024). However, the physiological context likely differed between tree groups. By the end of the flight period, surviving trees had largely expelled attacking beetles, whereas beetle-killed trees remained under sustained attack, with developing broods continuing to colonize the phloem. Thus, although both groups exhibited reduced monoterpene concentrations, the underlying mechanisms may differ, potentially reflecting recovery and downregulation of defense in surviving trees versus depletion of resources under continued biotic stress in beetle-killed trees. Further investigation is needed to disentangle these processes.

Although we did not detect a significant relationship between entry hole density and monoterpene concentration at the individual tree level, colonized trees exhibited a stronger induction compared to uncolonized trees (though the difference was marginally significant). This potentially supports the idea that greater attack severity can stimulate higher terpene production (Clark et al., 2012; Chiu and Bohlmann, 2022), though such patterns are not universally observed (Musso et al., 2023). Our findings also align with evidence that delayed responses to beetle attack increases the risk of tree mortality (Thompson et al., 2024). In beetle-killed trees, we observed no clear induction and monoterpene concentration remained close to baseline concentration throughout the growing season, with only a single time point showing a notable increase. This suggests that susceptible individuals lack the capacity for rapid induction of monoterpenes as well (Musso et al., 2023).

In this study, we show that both constitutive and early induced monoterpene production function as effective defenses against spruce bark beetle attack. We further demonstrate that constitutive monoterpene concentrations are strongly shaped by environmental conditions, particularly temperature and evaporative stress. Together, these findings help partially explain spatial patterns of forest mortality by suggesting that beetle success may be limited to colder, high-elevation environments where host defenses remain limited potentially due to shorter growing season. Our results indicate that bark beetle resistance treatments may be most effective when prioritized according to dominant environmental stressors. For Engelmann spruce, this may include low-temperature stress at high elevations and near timberline.

In the context of ongoing climate change, warming temperatures and shifting moisture regimes may alter these dynamics by enhancing tree defensive capacity in currently cold-limited environments, while potentially increasing stress at lower elevations. This could lead to a redistribution of beetle pressure across the landscape, with important implications for outbreak severity and spatial extent. However, such responses are likely to be species-specific. In the case of Engelmann spruce, susceptibility at higher elevations appears to be driven by cold, harsh climatic conditions, whereas in other conifer species, including many pines and spruce, susceptibility is more often associated with drought and heat stress. Accordingly, species-specific adaptive forest management strategies, such as prioritizing monitoring and intervention efforts in climatically vulnerable zones, will be critical for mitigating future bark beetle impacts.

Future studies should disentangle the relative contributions of environmental and genetic factors to resistance and determine whether Engelmann spruce susceptibility is primarily driven by cold stress at upper elevational limits, while at lower elevations and in drier climates it is reduced due to temperature and drought-induced upregulation of terpenes.

## Data Availability

The original contributions presented in the study are publicly available. This data can be found at (https://doi.org/10.5281/zenodo.19225955 / https://github.com/EhsanKhedive/khedive-engelmann-spruce-monoterpenes-data-2026).

## References

[B1] Akula R. Ravishankar G. A. (2011). Influence of abiotic stress signals on secondary metabolites in plants. Plant Signaling Behav. 6, 1720–1731. doi: 10.4161/psb.6.11.17613. PMID: 22041989 PMC3329344

[B2] Alexander R. R. Shepperd W. D. (1990). “ Picea engelmannii Parry ex Engelm. Engelmann spruce,” in Silvics of North America (Washington, DC, USA: U.S. Department of Agriculture, Forest Service), 1, 187–203.

[B3] Al-Khayri J. M. Rashmi R. Toppo V. Chole P. B. Banadka A. Sudheer W. N. . (2023). Plant secondary metabolites: The weapons for biotic stress management. Metabolites 13, 37. doi: 10.3390/metabo13060716. PMID: 37367873 PMC10302943

[B4] Baker G. Zhao S. Y. Klutsch J. G. Ishangulyyeva G. Erbilgin N. (2024). The legacy effect of mountain pine beetle outbreaks on the chemical and anatomical defences of surviving lodgepole pine trees. Metabolites 14, 24. doi: 10.3390/metabo14090472. PMID: 39330479 PMC11434468

[B5] Basile S. Stríbrska B. Kalyniukova A. Hradecky J. Synek J. Gershenzon J. . (2024). Physiological and biochemical changes of Picea abies (L.) during acute drought stress and their correlation with susceptibility to Ips typographus (L.) and I. duplicatus (Sahlberg). Front. For. Global Change 7. doi: 10.3389/ffgc.2024.1436110. PMID: 41890945

[B6] Baumer B. Udwin D. (2015). R markdown. WIREs Comput. Stat. 7, 167–177. doi: 10.1002/wics.1348. PMID: 41889077

[B7] Bentz B. J. Hansen E. M. Davenport M. Soderberg D. (2022). “ 2 - Complexities in predicting mountain pine beetle and spruce beetle response to climate change,” in Bark Beetle Management, Ecology, and Climate Change. Eds. Gandhi K. J. K. Hofstetter R. W. (San Diego, CA, USA: Academic Press), 31–54.

[B8] Beudert B. Bässler C. Thorn S. Noss R. Schröder B. Dieffenbach-Fries H. . (2015). Bark beetles increase biodiversity while maintaining drinking water quality. Conserv. Lett. 8, 272–281. doi: 10.1111/conl.12153. PMID: 41875165

[B9] Blodgett J. T. Stanosz G. R. (1998). Monoterpene and phenolic compound concentrations in water-stressed red pine inoculated with Sphaeropsis sapinea. Phytopathology 88, 245–251. doi: 10.1094/phyto.1998.88.3.245. PMID: 18944971

[B10] Bracalini M. Balacenoiu F. Panzavolta T. (2024). Forest health under climate change: Impact of insect pests. iForest - Biogeosc. For. 17, 295–299. doi: 10.3832/ifor4520-017. PMID: 17959540

[B11] Celedon J. M. Bohlmann J. (2019). Oleoresin defenses in conifers: Chemical diversity, terpene synthases and limitations of oleoresin defense under climate change. New Phytol. 224, 1444–1463. doi: 10.1111/nph.15984. PMID: 31179548

[B12] Chiu C. C. Bohlmann J. (2022). Mountain pine beetle epidemic: An interplay of terpenoids in host defense and insect pheromones. Annu. Rev. Plant Biol. 73, 475–494. doi: 10.1146/annurev-arplant-070921-103617. PMID: 35130442

[B13] Choudhary A. Senthil-Kumar M. (2022). Drought attenuates plant defence against bacterial pathogens by suppressing the expression of CBP60g/SARD1 during combined stress. Plant Cell Environ. 45, 1127–1145. doi: 10.1111/pce.14275. PMID: 35102557

[B14] Clark E. L. Huber D. P. W. Carroll A. L. (2012). The legacy of attack: Implications of high phloem resin monoterpene levels in lodgepole pines following mass attack by mountain pine beetle, Dendroctonus ponderosae Hopkins. Environ. Entomol. 41, 392–398. doi: 10.1603/EN11295. PMID: 22507014

[B15] Davis T. S. (2020). Toxicity of two Engelmann spruce (Pinaceae) monoterpene chemotypes from the southern Rocky Mountains to North American spruce beetle (Coleoptera: Scolytidae). Can. Entomol. 152, 790–796. doi: 10.4039/tce.2020.49

[B16] Davis T. S. Hansen E. M. (2018). An ordinal day model of spruce beetle trap capture phenology in northern Colorado. J. Appl. Entomol. 142, 277–281. doi: 10.1111/jen.12424. PMID: 41875165

[B17] Davis T. S. Horne F. B. Yetter J. C. Stewart J. E. (2018). Engelmann spruce chemotypes in Colorado and their effects on symbiotic fungi associated with the North American spruce beetle. J. Chem. Ecol. 44, 601–610. doi: 10.1007/s10886-018-0961-1. PMID: 29679267

[B18] Davis T. S. Rhoades P. R. Mann A. J. Griswold T. (2020). Bark beetle outbreak enhances biodiversity and foraging habitat of native bees in alpine landscapes of the southern Rocky Mountains. Sci. Rep. 10, 16400. doi: 10.1038/s41598-020-73273-z. PMID: 33009441 PMC7532438

[B19] de Groot M. Ogris N. Kobler A. (2018). The effects of a large-scale ice storm event on the drivers of bark beetle outbreaks and associated management practices. For. Ecol. Manage. 408, 195–201. doi: 10.1016/j.foreco.2017.10.035. PMID: 41903563

[B20] Dell I. H. Davis T. S. (2019). Effects of site thermal variation and physiography on flight synchrony and phenology of the North American spruce beetle (Coleoptera: Curculionidae, Scolytinae) and associated species in Colorado. Environ. Entomol. 48, 998–1011. doi: 10.1093/ee/nvz067. PMID: 31145459

[B21] Denham S. O. Coyle D. R. Oishi A. C. Bullock B. P. Heliövaara K. Novick K. A. (2019). Tree resin flow dynamics during an experimentally induced attack by Ips avulsus, I. calligraphus, and I. grandicollis. Can. J. For. Res. 49, 53–63. doi: 10.1139/cjfr-2018-0024. PMID: 36563491

[B22] Dymerski A. D. Anhold J. A. Munson A. S. (2001). Spruce beetle (Dendroctonus rufipennis) outbreak in Engelmann spruce (Picea engelmannii) in central Utah 1986-1998. West. N. Am. Nat. 61, 19–24.

[B23] Erbilgin N. Zanganeh L. Klutsch J. G. Chen S. H. Zhao S. Y. Ishangulyyeva G. . (2021). Combined drought and bark beetle attacks deplete non-structural carbohydrates and promote death of mature pine trees. Plant Cell Environ. 44, 3636–3651. doi: 10.1111/pce.14197. PMID: 34612515

[B24] Ferrenberg S. Kane J. M. Mitton J. B. (2014). Resin duct characteristics associated with tree resistance to bark beetles across lodgepole and limber pines. Oecologia 174, 1283–1292. doi: 10.1007/s00442-013-2841-2. PMID: 24305863

[B25] Gaylord M. L. Kolb T. E. Wallin K. F. Wagner M. R. (2007). Seasonal dynamics of tree growth, physiology, and resin defenses in a northern Arizona ponderosa pine forest. Can. J. For. Res. 37, 1173–1183. doi: 10.1139/X06-309. PMID: 36563491

[B26] Hart S. J. Veblen T. T. Eisenhart K. S. Jarvis D. Kulakowski D. (2014). Drought induces spruce beetle (Dendroctonus rufipennis) outbreaks across northwestern Colorado. Ecology 95, 930–939. doi: 10.1890/13-0230.1. PMID: 24933812

[B27] Hart S. J. Veblen T. T. Schneider D. Molotch N. P. (2017). Summer and winter drought drive the initiation and spread of spruce beetle outbreak. Ecology 98, 2698–2707. doi: 10.1002/ecy.1963, PMID: 28752623

[B28] Herms D. A. Mattson W. J. (1992). The dilemma of plants: To grow or defend. Q. Rev. Biol. 67, 283–335. doi: 10.1086/417659. PMID: 40881048

[B29] Johnson D. M. Haynes K. J. (2023). Spatiotemporal dynamics of forest insect populations under climate change. Curr. Opin. Insect Sci. 56, 6. doi: 10.1016/j.cois.2023.101020. PMID: 36906142

[B30] Karpov A. Pirtskhalava-Karpova N. Trubin A. Mezei P. Potterf M. Jakus R. (2024). Infestation patterns of two bark beetle species in multi-species coniferous forests on Kunashir Island in North Pacific Ocean region. For. Ecol. Manage. 558. doi: 10.1016/j.foreco.2024.121774. PMID: 41903563

[B31] Keefover-Ring K. Trowbridge A. Mason C. J. Raffa K. F. (2016). Rapid induction of multiple terpenoid groups by ponderosa pine in response to bark beetle-associated fungi. J. Chem. Ecol. 42, 1–12. doi: 10.1007/s10886-015-0659-6. PMID: 26662358

[B32] Klutsch J. G. Shamoun S. F. Erbilgin N. (2017). Drought stress leads to systemic induced susceptibility to a nectrotrophic fungus associated with mountain pine beetle in Pinus banksiana seedlings. PLoS One 12, 18. doi: 10.1371/journal.pone.0189203. PMID: 29216258 PMC5720781

[B33] Kolb T. Keefover-Ring K. Burr S. J. Hofstetter R. Gaylord M. Raffa K. F. (2019). Drought-mediated changes in tree physiological processes weaken tree defenses to bark beetle attack. J. Chem. Ecol. 45, 888–900. doi: 10.1007/s10886-019-01105-0. PMID: 31493165

[B34] Körner C. Basler D. Hoch G. Kollas C. Lenz A. Randin C. F. . (2016). Where, why and how? Explaining the low-temperature range limits of temperate tree species. J. Ecol. 104, 1076–1088. doi: 10.1111/1365-2745.12574. PMID: 41875165

[B35] Kurz W. A. Dymond C. C. Stinson G. Rampley G. J. Neilson E. T. Carroll A. L. . (2008). Mountain pine beetle and forest carbon feedback to climate change. Nature 452, 987–990. doi: 10.1038/nature06777. PMID: 18432244

[B36] Laxa M. Liebthal M. Telman W. Chibani K. Dietz K. J. (2019). The role of the plant antioxidant system in drought tolerance. Antioxidants (Basel) 8. doi: 10.3390/antiox8040094. PMID: 30965652 PMC6523806

[B37] López-Andújar Fustel T. Öhman K. Klapwijk M. Nordkvist M. Sängstuvall L. Lämås T. . (2024). Impact of management strategies on forest susceptibility to spruce bark beetle damage and potential trade-offs with timber production and biodiversity. For. Ecol. Manage. 563, 121964. doi: 10.1016/j.foreco.2024.121964. PMID: 41903563

[B38] Mageroy M. H. Christiansen E. Langstrom B. Borg-Karlson A. K. Solheim H. Bjorklund N. . (2020). Priming of inducible defenses protects Norway spruce against tree-killing bark beetles. Plant Cell Environ. 43, 420–430. doi: 10.1111/pce.13661. PMID: 31677172

[B39] Malone S. C. Thompson R. A. Chow P. S. de Oliveira C. R. Landhäusser S. M. Six D. L. . (2025). Water, not carbon, drives drought-constraints on stem terpene defense against simulated bark beetle attack in Pinus edulis. New Phytol. 245, 318–331. doi: 10.1111/nph.20218. PMID: 39462783 PMC11617656

[B40] Massey C. Wygant N. (1954). Biology and control of the Engelmann spruce beetle in Colorado (Washington, DC, USA: U.S. Department of Agriculture, Forest Service).

[B41] Morris J. L. Cottrell S. Fettig C. J. DeRose R. J. Mattor K. M. Carter V. A. . (2018). Bark beetles as agents of change in social–ecological systems. Front. Ecol. Environ. 16, S34–S43. doi: 10.1002/fee.1754. PMID: 41889077

[B42] Mullin M. Klutsch J. G. Cale J. A. Hussain A. Zhao S. Whitehouse C. . (2021). Primary and secondary metabolite profiles of lodgepole pine trees change with elevation, but not with latitude. J. Chem. Ecol. 47, 280–293. doi: 10.1007/s10886-021-01249-y. PMID: 33651224

[B43] Musso A. E. Fortier C. Huber D. P. W. Carroll A. L. Evenden M. L. (2023). Naive pine terpene response to the mountain pine beetle (Dendroctonus ponderosae) through the seasons. J. Chem. Ecol. 49, 299–312. doi: 10.1007/s10886-023-01418-1. PMID: 36929332

[B44] Netherer S. Lehmanski L. Bachlehner A. Rosner S. Savi T. Schmidt A. . (2024). Drought increases Norway spruce susceptibility to the Eurasian spruce bark beetle and its associated fungi. New Phytol. 242, 1000–1017. doi: 10.1111/nph.19635. PMID: 38433329

[B45] Ogwu M. C. Izah S. C. Joshua M. T. (2025). Ecological and environmental determinants of phytochemical variability in forest trees. Phytochem. Rev. 24, 5109–5137. doi: 10.1007/s11101-025-10066-0. PMID: 41894112

[B46] Ott D. S. Davis T. S. Mercado J. E. (2021). Interspecific variation in spruce constitutive and induced defenses in response to a bark beetle–fungal symbiont provides insight into traits associated with resistance. Tree Physiol. 41, 1109–1121. doi: 10.1093/treephys/tpaa170. PMID: 33450761

[B47] Pettit J. M. Voelker S. L. DeRose R. J. Burton J. (2020). Spruce beetle outbreak was not driven by drought stress: Evidence from a tree-ring iso-demographic approach indicates temperatures were more important. Global Change Biol. 26, 5829–5843. doi: 10.1111/gcb.15274. PMID: 32654317

[B48] Phillips M. A. Croteau R. B. (1999). Resin-based defenses in conifers. Trends Plant Sci. 4, 184–190. doi: 10.1016/S1360-1385(99)01401-6. PMID: 10322558

[B49] Plath E. Fischer K. (2024). Spruce dieback as chance for biodiversity: Standing deadwood promotes beetle diversity in post-disturbance stands in western Germany. J. Insect Conserv. 28, 525–537. doi: 10.1007/s10841-024-00571-6. PMID: 41894112

[B50] Potter K. M. Paschke J. L. (2024). “ Broad-scale patterns of insect and disease damage across the United States from the national Insect and Disease Survey 2022,” in Forest Health Monitoring: national status, trends, and analysis 2023 (Washington, DC, USA: U.S. Department of Agriculture, Forest Service).

[B51] Prescott C. E. (2022). Sinks for plant surplus carbon explain several ecological phenomena. Plant Soil 476, 689–698. doi: 10.1007/s11104-022-05390-9. PMID: 41894112

[B52] Qaderi M. M. Martel A. B. Strugnell C. A. (2023). Environmental factors regulate plant secondary metabolites. Plants-Basel 12, 27. doi: 10.3390/plants12030447. PMID: 36771531 PMC9920071

[B53] Rabeh K. Hnini M. Oubohssaine M. (2025). A comprehensive review of transcription factor-mediated regulation of secondary metabolites in plants under environmental stress. Stress Biol. 5, 15. doi: 10.1007/s44154-024-00201-w. PMID: 41894112

[B54] Raza M. M. Bebber D. P. (2022). Climate change and plant pathogens. Curr. Opin. Microbiol. 70, 102233. doi: 10.1016/j.mib.2022.102233. PMID: 36370642

[B55] R Core Team (2024). R: A language and environment for statistical computing. (Vienna, Austria: : R Foundation for Statistical Computing).

[B56] Rodman K. C. Andrus R. A. Butkiewicz C. L. Chapman T. B. Gill N. S. Harvey B. J. . (2021). Effects of bark beetle outbreaks on forest landscape pattern in the southern Rocky Mountains, U.S.A. Remote Sens. 13, 1089. doi: 10.3390/rs13061089. PMID: 41725453

[B57] Schiebe C. Hammerbacher A. Birgersson G. Witzell J. Brodelius P. E. Gershenzon J. . (2012). Inducibility of chemical defenses in Norway spruce bark is correlated with unsuccessful mass attacks by the spruce bark beetle. Oecologia 170, 183–198. doi: 10.1007/s00442-012-2298-8. PMID: 22422313

[B58] Seifert T. Breibeck J. Seifert S. Biber P. (2010). Resin pocket occurrence in Norway spruce depending on tree and climate variables. For. Ecol. Manage. 260, 302–312. doi: 10.1016/j.foreco.2010.03.024. PMID: 41903563

[B59] Sherriff R. L. Berg E. E. Miller A. E. (2011). Climate variability and spruce beetle (Dendroctonus rufipennis) outbreaks in south-central and southwest Alaska. Ecology 92, 1459–1470. doi: 10.1890/10-1118.1, PMID: 21870620

[B60] Siegert C. Clay N. Pace K. Vissa S. Hofstetter R. W. Leverón O. . (2024). Bark beetle-driven community and biogeochemical impacts in forest ecosystems: a review. Ann. Entomol. Soc Am. 117, 163–183. doi: 10.1093/aesa/saae009. PMID: 34249220

[B61] Singh V. V. Naseer A. Mogilicherla K. Trubin A. Zabihi K. Roy A. . (2024). Understanding bark beetle outbreaks: exploring the impact of changing temperature regimes, droughts, forest structure, and prospects for future forest pest management. Rev. Environ. Sci. Bio/Technol. 23, 257–290. doi: 10.1007/s11157-024-09692-5. PMID: 41894112

[B62] Stohlgren T. J. Bachand R. R. (1997). Lodgepole pine (Pinus contorta) ecotones in rocky mountain national park, Colorado, USA. Ecology 78, 632–641. doi: 10.2307/2266036. PMID: 39964225

[B63] Szulejko J. E. Kim K. H. (2014). Re-evaluation of effective carbon number (ECN) approach to predict response factors of ‘compounds lacking authentic standards or surrogates’ (CLASS) by thermal desorption analysis with GC–MS. Anal. Chim. Acta 851, 14–22. doi: 10.1016/j.aca.2014.08.033. PMID: 25440659

[B64] Taccoen A. Piedallu C. Seynave I. Gégout-Petit A. Gégout J. C. (2022). Climate change-induced background tree mortality is exacerbated towards the warm limits of the species ranges. Ann. For. Sci. 79, 22. doi: 10.1186/s13595-022-01142-y. PMID: 41896986

[B65] Thompson R. A. Malone S. C. Peltier D. Six D. Robertson N. Oliveira C. . (2024). Local carbon reserves are insufficient for phloem terpene induction during drought in Pinus edulis in response to bark beetle-associated fungi. New Phytol. 244, 654–669. doi: 10.1111/nph.20051. PMID: 39149848

[B66] Trowbridge A. M. Adams H. D. Collins A. Dickman L. T. Grossiord C. Hofland M. . (2021). Hotter droughts alter resource allocation to chemical defenses in pinon pine. Oecologia 197, 921–938. doi: 10.1007/s00442-021-05058-8. PMID: 34657177 PMC8591002

[B67] Vrba P. Beneš J. Čížek L. Filippov P. Faltýnek Fric Z. Hauck D. . (2024). Bark beetle outbreak and biodiversity in commercial spruce plantations: responses of four model groups. For. Ecol. Manage. 555, 121700. doi: 10.1016/j.foreco.2024.121700. PMID: 41903563

[B68] Wallis C. M. Huber D. P. W. Lewis K. J. (2011). Ecosystem, location, and climate effects on foliar secondary metabolites of lodgepole pine populations from central British Columbia. J. Chem. Ecol. 37, 607–621. doi: 10.1007/s10886-011-9958-8. PMID: 21537900

[B69] Yang L. Zhang X. Chen J. (2024). Winsorization greatly reduces false positives by popular differential expression methods when analyzing human population samples. Genome Biol. 25, 282. doi: 10.1186/s13059-024-03230-w. PMID: 39478636 PMC11523781

[B70] Zhao T. Krokene P. Hu J. Christiansen E. Bjorklund N. Langstrom B. . (2011). Induced terpene accumulation in Norway spruce inhibits bark beetle colonization in a dose-dependent manner. PLoS One 6, e26649. doi: 10.1371/journal.pone.0026649. PMID: 22028932 PMC3197568

