# Supplementary figures and images for: Climate indirectly modulates tree survival of spruce beetle attacks via effects on constitutive and induced secondary metabolites
Source: Front Plant Sci. 2026 Apr 22;17:1801237. doi: 10.3389/fpls.2026.1801237 (PMC13143916; doi:10.3389/fpls.2026.1801237)

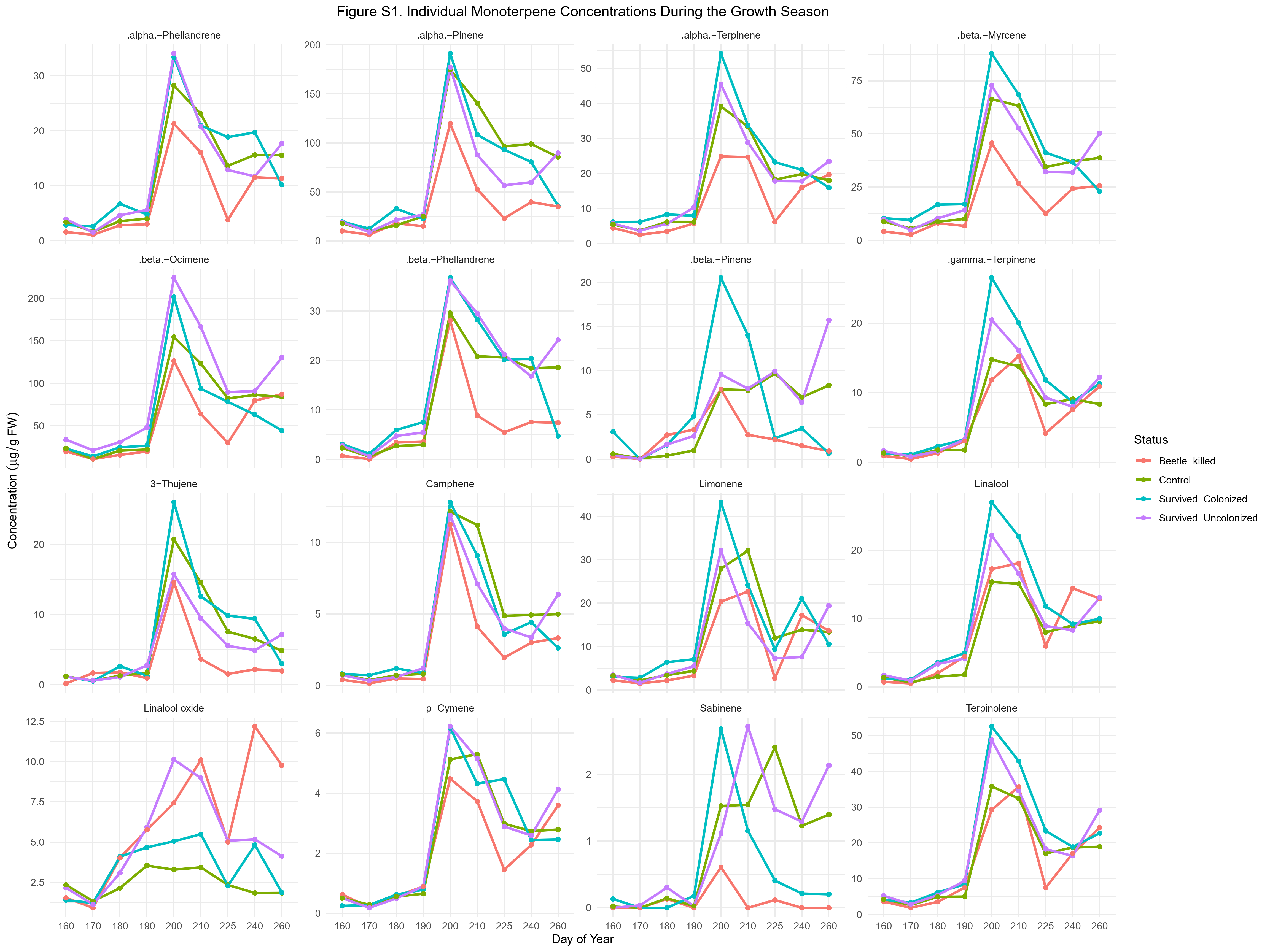

Supplement: Supplementary file 1 [file Image1.jpeg]

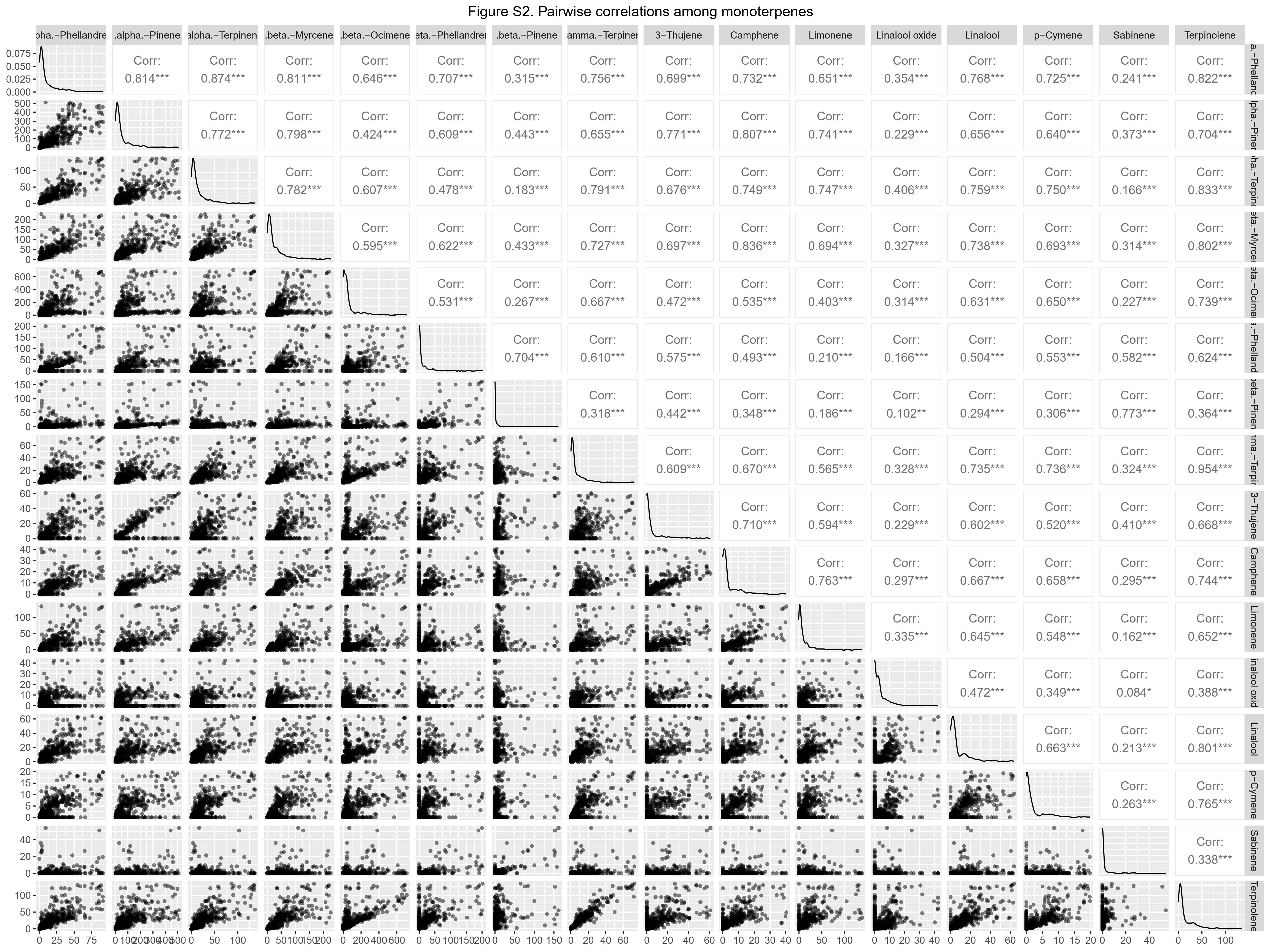

Supplement: Supplementary file 2 [file Image2.jpeg]
