# Supplementary material for: Climate indirectly modulates tree survival of spruce beetle attacks via effects on constitutive and induced secondary metabolites
Source: Front Plant Sci. 2026 Apr 22;17:1801237. doi: 10.3389/fpls.2026.1801237 (PMC13143916; doi:10.3389/fpls.2026.1801237)
Supplement: Supplementary file 3 [file Table1.docx]

Table S1. Results of complete block ANOVA of site effect on log of total monoterpene concentration

Model: log(Total monoterpenes) ~ Day of year + Site

Model coefficients

| Term | Estimate | SE | t | p-value |
| --- | --- | --- | --- | --- |
| (Intercept) | 3.92906955 | 0.1406436 | 27.9363575 | <0.001 |
| Day_of_year170 | -0.69410246 | 0.1651595 | -4.2026187 | <0.001 |
| Day_of_year180 | -0.11911757 | 0.1651595 | -0.7212274 | 0.4712 |
| Day_of_year190 | 0.19094424 | 0.1651595 | 1.1561201 | 0.2483 |
| Day_of_year200 | 2.01693944 | 0.1651595 | 12.2120694 | <0.001 |
| Day_of_year210 | 1.85469843 | 0.1687419 | 10.9913320 | <0.001 |
| Day_of_year225 | 1.18008834 | 0.1651595 | 7.1451430 | <0.001 |
| Day_of_year240 | 1.31660895 | 0.1651595 | 7.9717415 | <0.001 |
| Day_of_year260 | 1.33457042 | 0.1687866 | 7.9068494 | <0.001 |
| SiteCameron pass | 0.02844108 | 0.1234546 | 0.2303768 | 0.8179 |
| SiteEmpire | 0.28388155 | 0.1238074 | 2.2929283 | 0.0223 |
| SiteGrant | 0.31594859 | 0.1248997 | 2.5296175 | 0.0118 |
| SiteRabbit ears | 0.47128452 | 0.1241810 | 3.7951433 | <0.001 |

Model fit statistics

| Statistic | Value |
| --- | --- |
| Residual SE | 0.826 |
| R² | 0.562 |
| Adjusted R² | 0.55 |
| F statistic | 45.92 |
| Model p-value | < 0.001 |

Table S2. Results of log-linear ANOVA analysis of average annual temperature effect on total monoterpene concentration

Model: log(Total monoterpenes) ~ Day of year + Mean temperature

Model coefficients

| Term | Estimate | SE | t | p-value |
| --- | --- | --- | --- | --- |
| (Intercept) | 3.9019181 | 0.13603885 | 28.6823801 | <0.001 |
| Day_of_year170 | -0.6941025 | 0.16609164 | -4.1790330 | <0.001 |
| Day_of_year180 | -0.1191176 | 0.16609164 | -0.7171798 | 0.474 |
| Day_of_year190 | 0.1909442 | 0.16609164 | 1.1496318 | 0.251 |
| Day_of_year200 | 2.0169394 | 0.16609164 | 12.1435336 | <0.001 |
| Day_of_year210 | 1.8495784 | 0.16968354 | 10.9001637 | <0.001 |
| Day_of_year225 | 1.1800883 | 0.16609164 | 7.1050434 | <0.001 |
| Day_of_year240 | 1.3166090 | 0.16609164 | 7.9270030 | <0.001 |
| Day_of_year260 | 1.3385251 | 0.16967064 | 7.8889613 | <0.001 |
| Mean_temperature | 0.1294212 | 0.03596361 | 3.5986708 | <0.001 |

Model fit statistics

| Statistic | Value |
| --- | --- |
| Residual SE | 0.83 |
| R² | 0.554 |
| Adjusted R² | 0.545 |
| F statistic | 59.68 |
| Model p-value | < 0.001 |

Table S3. Results of log-linear ANOVA analysis of annual precipitation effect on total monoterpene concentration

Model: log(Total monoterpenes) ~ Day of year + Annual precipitation

Model coefficients

| Term | Estimate | SE | t | p-value |
| --- | --- | --- | --- | --- |
| (Intercept) | 4.4142953337 | 0.1884115874 | 23.4290013 | <0.001 |
| Day_of_year170 | -0.6941024648 | 0.1679246882 | -4.1334152 | <0.001 |
| Day_of_year180 | -0.1191175659 | 0.1679246882 | -0.7093511 | 0.4785 |
| Day_of_year190 | 0.1909442398 | 0.1679246882 | 1.1370826 | 0.2561 |
| Day_of_year200 | 2.0169394446 | 0.1679246882 | 12.0109763 | <0.001 |
| Day_of_year210 | 1.8422437953 | 0.1715397651 | 10.7394562 | <0.001 |
| Day_of_year225 | 1.1800883405 | 0.1679246882 | 7.0274857 | <0.001 |
| Day_of_year240 | 1.3166089501 | 0.1679246882 | 7.8404728 | <0.001 |
| Day_of_year260 | 1.3414084987 | 0.1715977245 | 7.8171695 | <0.001 |
| Precipitation | -0.0003410205 | 0.0001880277 | -1.8136715 | 0.0704 |

Model fit statistics

| Statistic | Value |
| --- | --- |
| Residual SE | 0.84 |
| R² | 0.544 |
| Adjusted R² | 0.535 |
| F statistic | 57.34 |
| Model p-value | < 0.001 |

Table S4. Results of log-linear ANOVA analysis of annual maximum vapor pressure deficit effect on total monoterpene concentration

Model: log(Total monoterpenes) ~ Day of year + Maximum VPD

Model coefficients

| Term | Estimate | SE | t | p-value |
| --- | --- | --- | --- | --- |
| (Intercept) | 3.5271954 | 0.2444501 | 14.4290999 | < 0.001 |
| Day_of_year170 | -0.6941025 | 0.1669404 | -4.1577860 | < 0.001 |
| Day_of_year180 | -0.1191176 | 0.1669404 | -0.7135335 | 0.47590 |
| Day_of_year190 | 0.1909442 | 0.1669404 | 1.1437869 | 0.25335 |
| Day_of_year200 | 2.0169394 | 0.1669404 | 12.0817933 | < 0.001 |
| Day_of_year210 | 1.8465446 | 0.1705445 | 10.8273466 | < 0.001 |
| Day_of_year225 | 1.1800883 | 0.1669404 | 7.0689199 | < 0.001 |
| Day_of_year240 | 1.3166090 | 0.1669404 | 7.8867004 | < 0.001 |
| Day_of_year260 | 1.3378675 | 0.1705389 | 7.8449417 | < 0.001 |
| Maximum_VPD | 0.6982703 | 0.2403903 | 2.9047357 | 0.00386 |

Model fit statistics

| Statistic | Value |
| --- | --- |
| Residual SE | 0.835 |
| R² | 0.55 |
| Adjusted R² | 0.54 |
| F statistic | 58.59 |
| Model p-value | < 0.001 |

Table S5. Results of complete block ANOVA analysis of average annual temperature effect on induced fold change monoterpene concentration

Table S5-1: Flight period induced response

Model: Fold change ~ Day of year + Mean temperature + Status

Model coefficients

| Term | Estimate | SE | t | p-value |
| --- | --- | --- | --- | --- |
| (Intercept) | 0.64839774 | 0.24570432 | 2.6389350 | 0.00924 |
| Day_of_year180 | 0.17348219 | 0.19279031 | 0.8998491 | 0.36971 |
| Day_of_year190 | 0.31702917 | 0.19279031 | 1.6444248 | 0.10229 |
| Mean_temperature | 0.09237251 | 0.07371942 | 1.2530282 | 0.21224 |
| StatusSurvived-Colonized | 0.57343450 | 0.26596326 | 2.1560666 | 0.03275 |
| StatusSurvived-Uncolonized | 0.41168840 | 0.22621343 | 1.8199114 | 0.07086 |

Model fit statistics

| Statistic | Value |
| --- | --- |
| Residual SE | 0.959 |
| R² | 0.069 |
| Adjusted R² | 0.037 |
| F statistic | 2.12 |
| Model p-value | 0.065957 |

Table S5-2: Post-flight period induced response

Model: Fold change ~ Day of year + Mean temperature + Status

Model coefficients

| Term | Estimate | SE | t | p-value |
| --- | --- | --- | --- | --- |
| (Intercept) | 1.27077695 | 0.24975003 | 5.0881953 | <0.001 |
| Day_of_year210 | -0.57446271 | 0.23428551 | -2.4519771 | 0.0149 |
| Day_of_year225 | -0.52688865 | 0.23173488 | -2.2736700 | 0.0239 |
| Day_of_year240 | -0.46169135 | 0.23051795 | -2.0028434 | 0.0463 |
| Day_of_year260 | -0.32329259 | 0.23051833 | -1.4024594 | 0.1621 |
| Mean_temperature | 0.03789135 | 0.06938999 | 0.5460637 | 0.5855 |
| StatusSurvived-Colonized | 0.37506078 | 0.24944224 | 1.5035977 | 0.1340 |
| StatusSurvived-Uncolonized | 0.05410300 | 0.21021082 | 0.2573749 | 0.7971 |

Model fit statistics

| Statistic | Value |
| --- | --- |
| Residual SE | 1.147 |
| R² | 0.047 |
| Adjusted R² | 0.019 |
| F statistic | 1.65 |
| Model p-value | 0.12175 |

Table S6. Results of ANOVA analysis on Entry hole density of trees in each site

Model: Entry holes ~ Site

Model coefficients

| Term | Estimate | SE | t | p-value |
| --- | --- | --- | --- | --- |
| (Intercept) | 23.134000 | 5.546714 | 4.1707580 | <0.001 |
| SiteAlma | -1.614000 | 9.057745 | -0.1781900 | 0.860 |
| SiteEmpire | 8.377429 | 8.643929 | 0.9691691 | 0.339 |
| SiteRabbit ears | 9.146000 | 8.059188 | 1.1348538 | 0.264 |
| SiteCameron pass | 5.971800 | 7.844237 | 0.7612977 | 0.451 |

Model fit statistics

| Statistic | Value |
| --- | --- |
| Residual SE | 17.54 |
| R² | 0.062 |
| Adjusted R² | -0.04 |
| F statistic | 0.61 |
| Model p-value | 0.6596 |

Table S7. Result of Complete block ANOVA analysis of fold change concentration of monoterpenes in trees with different status

Model: Fold change ~ Day of year + Status

Model coefficients

| Term | Estimate | SE | t | p-value |
| --- | --- | --- | --- | --- |
| (Intercept) | 0.85318033 | 0.1849380 | 4.61333125 | <0.001 |
| Day_of_year170 | 0.09443830 | 0.2087563 | 0.45238544 | 0.6512 |
| Day_of_year180 | 0.26677397 | 0.2076979 | 1.28443260 | 0.1997 |
| Day_of_year190 | 0.41032095 | 0.2076979 | 1.97556608 | 0.0488 |
| Day_of_year200 | 0.34313807 | 0.2076979 | 1.65210168 | 0.0992 |
| Day_of_year210 | -0.23053977 | 0.2121733 | -1.08656376 | 0.2778 |
| Day_of_year225 | -0.18019937 | 0.2098560 | -0.85868117 | 0.3910 |
| Day_of_year240 | -0.11875247 | 0.2087648 | -0.56883386 | 0.5698 |
| Day_of_year260 | 0.01814553 | 0.2087648 | 0.08691854 | 0.9308 |
| StatusSurvived-Colonized | 0.47373862 | 0.1654708 | 2.86297340 | 0.0044 |
| StatusSurvived-Uncolonized | 0.25262936 | 0.1371343 | 1.84220413 | 0.0661 |

Model fit statistics

| Statistic | Value |
| --- | --- |
| Residual SE | 1.038 |
| R² | 0.06 |
| Adjusted R² | 0.039 |
| F statistic | 2.77 |
| Model p-value | 0.0025451 |

Table S8. Result of logistic regression showing monoterpene concentration fold change effect on tree survival

Table S8-1: Flight period survival analysis

Model: Survival ~ Fold change × Day of year

Model coefficients

| Term | Estimate | SE | t | p-value |
| --- | --- | --- | --- | --- |
| (Intercept) | 0.296498 | 0.4263542 | 0.6954264 | 0.4868 |
| Fold_change:Day_of_year160 | 1.450912 | 0.5994670 | 2.4203368 | 0.0155 |
| Fold_change:Day_of_year170 | 1.500118 | 0.5909815 | 2.5383508 | 0.0111 |
| Fold_change:Day_of_year180 | 1.036024 | 0.4447110 | 2.3296562 | 0.0198 |
| Fold_change:Day_of_year190 | 1.117307 | 0.4774388 | 2.3402100 | 0.0193 |

Model fit statistics

| Statistic | Value |
| --- | --- |
| Null deviance | 175.167 |
| Null DF | 197 |
| Deviance | 161.855 |
| Residual DF | 193 |
| AIC | 171.86 |

Table S8-2: Post-flight period survival analysis

Model: Survival ~ Fold change × Day of year

Model coefficients

| Term | Estimate | SE | t | p-value |
| --- | --- | --- | --- | --- |
| (Intercept) | 1.44406923 | 0.2668744 | 5.4110439 | <0.001 |
| Fold_change:Day_of_year200 | -0.13274090 | 0.2374677 | -0.5589852 | 0.576 |
| Fold_change:Day_of_year210 | 0.47869035 | 0.5266479 | 0.9089381 | 0.363 |
| Fold_change:Day_of_year225 | 0.37373247 | 0.4333400 | 0.8624463 | 0.388 |
| Fold_change:Day_of_year240 | 0.37140145 | 0.4671859 | 0.7949758 | 0.427 |
| Fold_change:Day_of_year260 | 0.08845552 | 0.3268204 | 0.2706548 | 0.787 |

Model fit statistics

| Statistic | Value |
| --- | --- |
| Null deviance | 215.904 |
| Null DF | 238 |
| Deviance | 212.79 |
| Residual DF | 233 |
| AIC | 224.79 |

Table S9. Result of logistic regression test of speed and magnitude of monoterpene induction on survival

Model: Survival ~ Speed × Magnitude

Model coefficients

| Term | Estimate | SE | t | p-value |
| --- | --- | --- | --- | --- |
| (Intercept) | -1.58186255 | 3.585130 | -0.441228753 | 0.659 |
| Speed170 | 1.47019539 | 4.859793 | 0.302522228 | 0.762 |
| Speed180 | 7.39629140 | 5.288223 | 1.398634599 | 0.162 |
| Speed190 | -78.81862861 | 22,398.176558 | -0.003518975 | 0.997 |
| Speed200 | 17.96976229 | 10,754.012966 | 0.001670982 | 0.999 |
| Speed210 | 18.90623494 | 10,754.013037 | 0.001758063 | 0.999 |
| Speed260 | 19.07642283 | 10,754.013108 | 0.001773889 | 0.999 |
| Magnitude | 3.06642254 | 3.022437 | 1.014552957 | 0.310 |
| Speed170:Magnitude | -0.06885605 | 5.137682 | -0.013402164 | 0.989 |
| Speed180:Magnitude | -8.54513331 | 4.929516 | -1.733462844 | 0.083 |
| Speed190:Magnitude | 100.63843035 | 26,412.072372 | 0.003810319 | 0.997 |
| Speed200:Magnitude |  |  |  | NA |
| Speed210:Magnitude |  |  |  | NA |
| Speed260:Magnitude |  |  |  | NA |

Model fit statistics

| Statistic | Value |
| --- | --- |
| Null deviance | 39.88 |
| Null DF | 47 |
| Deviance | 25.881 |
| Residual DF | 37 |
| AIC | 47.881 |

* NA in some rows is the result of having only one replicates in those factor levels
